# Our Schoolyard Infrastructure Just Isn’t Cutting It: Play *Is* Public Health

**DOI:** 10.5888/pcd22.250121

**Published:** 2025-07-24

**Authors:** Kylie Wilson, Marissa Schulke

**Affiliations:** 1 Arizona State University, College of Health Solutions, Phoenix

## Introduction

Today, young people face increasing health challenges, many of which are due to declining opportunities for physical exercise and the interpersonal connections fostered by active play (1). Physical activity, which can prevent obesity and related chronic diseases, is at alarmingly low levels — only 16% of adolescents and 26% of children in the US meet the Healthy People 2030 physical activity guidelines (2). Simultaneously, young people are experiencing declining social connections, which support psychosocial health (3). Given that most people spend nearly 2 decades of their formative years in prekindergarten-to-grade 12 schools, these schools are critical settings for shaping lifelong health behaviors and attitudes. However, one of the most visible current challenges to promoting youth play for health is the condition of our school infrastructure (4), particularly outdoor spaces where active play most often occurs (5). Although infrastructure problems are related not only to schoolyards, these settings are particularly positioned to foster play, which has profound benefits for both acute and chronic health.

We, as physical activity and public health scholars, conduct community-based research, often in schools. When on a cool 101°F day (for Arizona!) we were collecting recess data at a local school, a new team member asked, “How is *that* ADA compliant?” pointing at a jutting asphalt lip outside the door to the multipurpose room, the access point from indoors to the schoolyard. The observation wasn’t surprising. We repeatedly encounter outdated, inaccessible, weather-vulnerable schoolyards that fail to meet the federal guidelines and the needs of students and their communities.

Despite longstanding recognition of the importance of safe and engaging school environments for public health, infrastructure investments haven’t kept pace. We highlight 2 persistent challenges from our experience that demonstrate how current schoolyard infrastructure fails to address accessibility and extreme weather conditions. We propose solutions to initiate community-driven change to play spaces to promote better health.

### Accessibility

Accessibility for all children, particularly children with disabilities, is both a public health priority and a persistent challenge (6–8). The Americans with Disabilities Act (ADA) mandates that public schools adhere to accessibility guidelines, such as accessible routes to play components (eg, slides, swings) (9,10). However these requirements are the bare minimum, and many schoolyards nonetheless remain inaccessible to students with disabilities, preventing full participation in recess, physical education, and outdoor activities. For example, schools might have a rubberized pathway to a play structure, per ADA requirements (10), but the structure may remain inaccessible to students with mobility disabilities if the pathway ends with stairs. Even when play spaces include accessible pathways, few include appropriate surfacing, and fewer still have universal and adaptive play equipment (eg, wheelchair ramps, adapted mobility swings) (6). When students with mobility disabilities have to navigate uneven terrain and structural barriers, their ability to interact and play with peers is hindered.

Infrastructure challenges are even greater for some subgroups. In a prior study, we found playgrounds and playfields in schools in low-income neighborhoods were 3 to 7 times less accessible for students with disabilities than for those without disabilities (11). This is especially alarming given that people with disabilities are one of the largest marginalized groups worldwide (12). Inadequate funding and policy implementation are a few ways these inequities continue to increase gaps in accessibility and social connectivity.

Many schools simply lack financial resources to retrofit or renovate their infrastructure, especially those in low-income, high-need areas. The US spends approximately $18,500 per pupil from local, state, and federal funding, about 10% of which is partitioned for school renovations and alterations (13). Most schools make indoor renovations while neglecting outdoor areas; however, improving playgrounds and making them better accessible can enhance children’s physical, social-emotional, and mental health — a necessary priority to overcome health disparities.

Resources are available to address infrastructure issues. For example, the ADA Checklist (14) and Child Community Health Inclusion Index (15) are useful tools to identify areas of need and can be used to seek funding and actualize change through state grants, local bonds, and community partnerships. Involving young people themselves in the co-creation of their playground redesign is also critical to creating universally enjoyable, suitable, and accessible spaces.

Developing schoolyards that accommodate students with disabilities is not just about meeting federal requirements. Our current infrastructure prioritizes play among students without disabilities while the physical, social-emotional, and mental health of students with disabilities suffers. This lack of attention demands that policymakers, schools, and communities reconceptualize how play-for-all looks. What child is excluded when school playgrounds are universally designed?

### Extreme weather

A second major infrastructure shortfall is the failure to build schoolyards with consideration for regional weather. Extreme weather, including excessive heat, cold, flooding, and storms, has historically disrupted outdoor school-based play. As infrastructure ages (4) and these extremes either persist or become more intense or frequent (16), lack of supportive infrastructure hampers our ability to provide consistent, safe play spaces (5), compounding the decline in young people’s play. 

For example, in heat-prone regions like Arizona, where our research is based, schoolyards often lack adequate protection against extreme heat and sun exposure (17). Unshaded, heat-absorbing surfaces like asphalt, concrete, and metal, which are common in schoolyards, can be dangerously hot, and most elementary schools in the Southwest don’t have indoor gymnasiums. Although research continues to explore the acute and chronic health problems associated with overexposure to extreme heat and sun, such as exertional heat stroke risk and skin cancer, we already know that prolonged exposure increases the risk of dehydration and heat-related illnesses, particularly among people with preexisting health conditions (18).

Problems related to extreme heat are pervasive. For example, in the fall semester of 2023 in Maricopa County, where more than 60% of the Arizona population resides, our own pilot data show that elementary schools canceled or modified 5 or more weeks of recess because of heat. As with accessibility concerns, schools in underserved communities often lack the financial capacity to implement heat-mitigation strategies such as shade structures, tree planting, or use of heat-resistant materials. These disparities contribute to systemic health inequities, because young people in low-income areas have limited access to safe spaces for physical activity and outdoor play even before adaptations for extreme weather can be considered (19).

But it’s not all bad news. Awareness is growing, and communities are taking action. Schools, governments, and researchers are bolstering collaborative work toward weather-ready infrastructure that ensures year-round access to safe play opportunities. A promising example is the HeatReady Schools initiative, a part of the HeatReady Program developed by the City of Phoenix and researchers at Arizona State University to “identify, prepare for, mitigate, track, and respond to the negative impacts of schoolground heat” (20). Passive cooling strategies such as increasing shade through native tree canopy coverage and installing shade structures can significantly reduce heat risks (17). Given that many schools lack viable indoor play alternatives for inclement weather, such adaptations are critical to sustaining movement opportunities. Funding sources like state grants, environmental resilience programs, and local partnerships can help schools implement these improvements (eg, American Forests, Trees Matter, American Academy of Dermatology), but continued attention to the matter will be paramount as weather patterns and physical activity needs continue to shift.

## Call to Action

Schoolyard infrastructure isn’t just about aesthetics and convenience. It’s a critical public health issue. Accessible and weather-ready schoolyards are only 2 examples highlighting the importance of ensuring that all students, regardless of ability or background, have safe opportunities to play. Although the obstacles are real, so are opportunities for action.

Change requires more than top-down mandates, although these are influential in their own right. School boards, where key infrastructure decisions are made, often lack momentum without significant community pressure. Grassroots movements are essential in generating momentum and co-creating. When schools, parents, local governments, and allies unite, they amplify the voices of those most affected — students, families, and community members who intimately understand their schools’ needs. Collective effort can help initiate policies, secure funding, and push for necessary change. Local advocates can partner with school boards to conduct accessibility audits, engage in weather resilience planning, and explore funding opportunities, keeping community needs at the forefront. By rallying together, communities can make meaningful improvements that benefit the entire community.

Researchers are also uniquely positioned to provide supporting evidence urging better schoolyard infrastructure. By conducting studies demonstrating long-term health, educational, and economic benefits, researchers can inform policymakers and communities about the true cost of inaction. Researchers can work with schools and communities to co-design and evaluate pilot programs, monitor the success of implemented strategies, assist with grant writing to secure funding, and co-develop best practices that prioritize health. Partnering with local advocates ensures data-driven, evidence-based support for grassroots movements, strengthening messaging that resonates with decision makers. Embedding research into communities ensures findings go beyond academic journals and drive effective change.

The cracks in schoolyard infrastructure have been widening for far too long, and we’re now, unsurprisingly, seeing the consequences of poor planning, limited forward-thinking, and inaction. Neglecting these spaces perpetuates health inequities and limits opportunities for healthy behaviors. A well-designed, accessible, inclusive, and weather-ready schoolyard is not a luxury. It’s the bare minimum in fostering a healthier generation. This issue remains central to our research, and we invite you to join us in ensuring that all students, at every school, in every community, can grow, learn, and play in environments that support lifelong health and resilience.
